# Leveraging three-tier deep learning model for environmental cleaner plants production

**DOI:** 10.1038/s41598-023-43465-4

**Published:** 2023-11-09

**Authors:** Zahraa Tarek, Mohamed Elhoseny, Mohamemd I. Alghamdi, Ibrahim M. EL-Hasnony

**Affiliations:** 1 Faculty of Computers and Information Science, Mansoura University, Mansoura, Egypt; 2 College of Computing and Informatics, University of Sharjah, Sharjah, United Arab Emirates; 3 Department of Computer Science, Al-Baha University, Al Bahah, Kingdom of Saudi Arabia

**Keywords:** Engineering, Energy infrastructure

## Abstract

The world's population is expected to exceed 9 billion people by 2050, necessitating a 70% increase in agricultural output and food production to meet the demand. Due to resource shortages, climate change, the COVID-19 pandemic, and highly harsh socioeconomic predictions, such a demand is challenging to complete without using computation and forecasting methods. Machine learning has grown with big data and high-performance computers technologies to open up new data-intensive scientific opportunities in the multidisciplinary agri-technology area. Throughout the plant's developmental period, diseases and pests are natural disasters, from seed production to seedling growth. This paper introduces an early diagnosis framework for plant diseases based on fog computing and edge environment by IoT sensors measurements and communication technologies. The effectiveness of employing pre-trained CNN architectures as feature extractors in identifying plant illnesses has been studied. As feature extractors, standard pre-trained CNN models, AlexNet are employed. The obtained in-depth features are eliminated by proposing a revised version of the grey wolf optimization (GWO) algorithm that approved its efficiency through experiments. The features subset selected were used to train the SVM classifier. Ten datasets for different plants are utilized to assess the proposed model. According to the findings, the proposed model achieved better outcomes for all used datasets. As an average for all datasets, the accuracy of the proposed model is 93.84 compared to 85.49, 87.89, 87.04 for AlexNet, GoogleNet, and the SVM, respectively.

## Introduction

In the global economy, agriculture is crucial. The agriculture system will face more strain as the human population grows and the COVID-19 pandemic takes hold. Agri-technology has developed as a novel scientific area that employs data-intensive techniques to boost agricultural output while reducing the environmental impact. In contemporary agricultural activities, data is created by a variety of sensors that give a better understanding of the operational surroundings (weather conditions, dynamic crop, and soil) as well as the operation itself (data from machines), resulting in more precise and quicker decision-making^1^. Conservation agriculture has long been regarded as an effective and ecologically beneficial management strategy to boost agricultural yields. In addition, measuring the total impact of conservation agriculture on crop output amelioration by taking the average of the entire dataset is not unfamiliar. Nevertheless, the influence of conservation agriculture on yielding cleaner output should be examined^2^.

Plant disease and pest identification are critical research areas in the realm of machine learning. To determine whether or not a plant image contains diseases or pests, machine vision equipment is used^3^. Are these types of detection systems needed? A vital food security hazard is plant disease. Agricultural and population growth are affected by plant diseases, as is the economy. Disease control, food safety, and anticipated loss of income need an automated and exact estimation of plant disease gravity. Plant diseases must thus be identified and treated at an early stage. Non-expert farmers, on the other hand, are frequently oblivious to non-native illnesses, necessitating consultation with experts to determine whether there are any strange symptoms or appearances on their crops. A farmer may have to travel vast distances to consult with an expert, which is costly and time-consuming. To automate the process of identifying plant illnesses, these difficulties motivate research and expansion in this area. The essential requirement for a plant disease diagnosis model that can operate in an Internet of Things (IoT) environment with minimal processing capabilities is very important^4^.

Already, machine vision is being used in agriculture to detect plant diseases and pests. While artificial intelligence (AI) is still a long way from being widely deployed, the technology has tremendous development potential and application value^5^. Plant diseases have been classified and identified using machine learning (ML) models. Nonetheless, with improvements in deep learning (DL), this field of research looks to offer enormous promise for greater accuracy. When it comes to planting disease detection, multiple DL structures have been developed or modified, as have many visualization methodologies. Various performance measurements are also used to evaluate these architectures and techniques^6^.

As a result, several researchers have sought to build robust plant disease detection systems that require a high number of disease-infected specimens to be successful. In the past, collecting such a vast number of samples was difficult. Thanks to the Internet of Things, we can now gather and diagnose diseases within the human body! As part of the Plantvillage datasets, there are a lot of photos of corpses with various diseases. Because it is well-labeled and extensively utilized, this dataset has been used in several plant disease detection studies. To maximize their harvests, the farmers also want an easy-to-use detection system that they can use on their phones to identify plant diseases and remove them early. Using image processing methods, Plant disease farmers and researchers may be able to diagnose plant ailments more precisely. Image processing techniques for detecting sickness can also yield satisfactory results, but they require human intervention for other detection and analysis^7–9^.

According to these challenges, we aim to improve the quality of the product and arrive at cleaner production. For plant disease and pest identification using machine vision, the emphasis has switched from standard machine learning and image processing approaches to deep learning techniques in fog environments, which have handled complex previously unsolvable issues. The paper contribution handles four-folds. The first fold is using IoT sensors to generate data and images; there are many sensors used in this field, such as soil moisture, humidity, and temperature, light-dependent resistors, water level, relay module, analog extender, and buzzer ESP 8266. These images were preserved for ten economically and environmentally beneficial plants. Leaf pictures of these plants in ideal and dire circumstances have been collected and dispersed across two categories. In this paper, datasets that focus on plants with significant ecological and economic benefits to their ecosystems are examined. As a result, ten plants, popularly known as Arjun, Mango, Guava, Saptaparni, Jamun, Bael, Sukh Chain, Jatropha, Pomegranate, Basil, Chinar, and Lemon, have been picked. To name just a few, some of these plants have high medicinal value; others are popular for their fruits, and the vast majority are environmentally and economically significant.

Algorithms and models based on deep learning must be successfully integrated with agricultural and plant protection experience to fully exploit AL and ML's potential in the second fold. Three deep learning models applied for plant disease detection are AlexNet, one of the most widely used neural network designs nowadays, GoogleNet, one of the most significant advances in the domain of Neural Networks, notably for CNNs the support vector machine (SVM). These models are utilized to the deep extracted features generated by the pre-trained CNN layers, AexNet, and we extracted the (fc7) layer as our feature extracting layer. After feeding images into that layer, we can receive features of the images from it. After having all the features, we could use them for training the classifiers. Then, a comparative study among the three models is conducted to show the accuracy of the three models.

The third fold is using a fog environment for computing all necessary tasks of image preprocessing, visualization, monitoring, and local decision support systems for detection and prediction tasks. As a new way of extending and assisting cloud computing, Fog Computing is a rapidly evolving technology. Its proximity to edge users, openness, and mobility, make fog computing platforms ideal for providing services to users quickly and improving the QoS (Quality of Service) of Internet of Things devices. A customer application based on IoT involving real-time activities in agriculture is increasingly reliant on this method^10^.

Lastly, developing a novel version of the grey wolf optimization algorithm (GWO) for selecting the important features to feed to the classifiers. This process is very important to select the relevant features to accelerate the prediction models with fair accuracy. The selected features are fed to the SVM and compared to the standard model, which used all the features from AlexNet.

The remainder of the paper is structured as follows: “Related work” provides some studies about the recent work. An overview of the basic concepts and methods utilized in this paper is presented in “Methods and overviews”. “Proposed methodology” provides the suggested methodology in detail. The experiment setting and results are shown in “Experimental results and discussion”. “Conclusion and future work” concludes with a look at what's next.

## Related work

Abbas et al.^11^, presented a technique based on deep learning for tomato disease diagnosis. To categorize tomato leaf pictures into ten disease categories, the DenseNet121 approach was trained on real and synthetic images using transfer learning. The suggested approach attained an accuracy of 97.11%, 98.65%, and 99.51% for the classification of leaf images into 10 classes, 7 classes, and 5 classes, respectively.

Thenmozhi and Reddy^12^, proposed a powerful CNN approach, and transfer learning is being applied to achieve the best or a desired performance of the pre-training model. Three public insect datasets were used to classify insect species, with accuracy rates of 96.75 percent, 97.47 percent, and 95.97 percent, respectively. Wiesner-Hanks et al.^13^, utilized community data for training a CNN, and nutrition the output into a conditional random field (CRF) to divide pictures into non-lesion and lesion areas with an accuracy of 0.9979 and F1 score of 0.7153.

Too et al.^14^, utilized DenseNets, which have a propensity to always progress in accuracy as the number of iterations increases, with no evidence of performance decay or overfitting. For the classification of plant disease, an accuracy score of 99.75% was achieved. Chen et al.^15^, presented CNN architecture depended on a gliding window to construct a structure for location regression calculation and recognition of pests’ species and plant diseases, feature fusion, characteristics automatic learning, and the identification rate of 38 frequent symptoms was 50–90%. Zhou et al.^16^, demonstrated a rapid approach for the detection of rice diseases founded on the combination of Faster R-CNN and FCM-KM. The sheath blight, bacterial blight, and detection accuracy, and rice blast time were 98.26 percent/0.53 s, 97.53 percent/0.82 s, and 96.71 percent/0.65 s respectively, based on the application results of 3010 images.

Sethy et al.^17^ presented 5932 on-field pictures of 4 different kinds of rice leaf illnesses: brown spot, bacterial blight, tungro, and blast. Furthermore, the effectiveness of eleven CNN architectures in the deep feature with SVM and the transfer learning approach was assessed. According to the experimental findings, the deep feature of ResNet50 with SVM outperforms transfer learning equivalent in classification. Deep learning-based methods for identifying illnesses and pests in rice plant pictures have been developed by Rahman et al.^18^. A two-stage tiny CNN design was developed, and it was compared to SqueezeNet, NasNet Mobile, and MobileNet. The simulation findings demonstrated that the suggested framework could attain the necessary accuracy of 93.3%.

Guo et al.^19^ presented a mathematical model based on deep learning for the recognition of plant disease and detection. The model was tested for illnesses such as rust diseases, black rot, and bacterial plaque. The results indicated that the accuracy of the model is 83.57%, which is greater than the previous technique, decreasing the impact of illness on agricultural productivity and being beneficial to agriculture's long-term improvement. Atila et al.^20^, presented the EfficientNet model for the plant leaf disease classification, and the performance of the model was compared to existing previous deep learning techniques. The experimental findings revealed that the B4 and B5 approaches of the EfficientNet attained the greatest rates in the original and enhanced datasets, with the accuracy of 99.91% and 99.97%, and precision of 98.42% and 99.39% respectively. There are many studies for plan disease prediction as in Refs.^7–9,21^.

Table 1 summarizes the role of ML/DL in agriculture for plant diseases classification using accuracy measurement as mentioned by many authors. It is observable that most of the recent works use the PlantVillage dataset and deploying a set of pre-trained CNN models. In this paper, new datasets have been used for testing our proposed architecture for plant disease classification.

**Table 1:** ML/DL for plant disease detection.

Author	Dataset	Method	Result
^22^	Banana leaf images obtained from banana field	ResNet-152	99.2%
^23^	PlantVillage	VGG-19	98.3%
^24^	PlantVillage	Multi-Scale AlexNet	92.7%
^25^	PlantVillage	ResNet34	99.7%
^26^	PlantVillage	CNN	91.2%,
^27^	Collected data from field	Custom-Net	98.78%
^28^	6 Common cucumber leaf diseases taken in field	GPDCNN	95.18%
^29^	PlantVillage	CAE	86.78%
^30^	PlantVillage	CAE + CNN	98.38%

The next section handles an overview of the problem statement and the used methods in this paper.

## Methods and overviews

### Overvies

Climate change, population expansion, and food security concerns have pushed the sector to explore more creative ways to agricultural yield protection and improvement. As a result, artificial intelligence (AI) is progressively developing as a component of the industry's technical growth^31^. Popular applications of traditional machine learning algorithms in agriculture are:

- Recognition/harvesting of vegetables and fruits.
- Plant disease classification/pest detection.
- Crop/weed discernment and classification.
- Plant/leaves recognition and classification.
- Land cover classification.

In comparison to the defined segmentation, detection, and classification tasks in computer vision, the criteria for detecting pests and plant diseases are quite broad. Its needs may be classified into three categories: what, where, and how. Even though, the fact that the function needs and aims of the 3 phases of plant disease and pest detection are distinct, the three stages are mutually inclusive^32^. The classification job in computer vision is represented by "what" in the first step. Classification defines the image globally using feature expression and then decides if the image contains a certain type of object using the classification process. While structure learning is the primary research path in object detection, feature expression is the primary research path in classification tasks.

Machine learning (ML) has developed alongside high-performance computation and big data technologies to open up new avenues for unraveling, quantifying, and comprehending data-intensive processes in agricultural operational contexts. ML offers machines the capacity to learn without being precisely programmed^33^. Convolutional neural networks (CNNs) are more complex to construct than traditional neural networks, but they are simpler to utilize. It is not required to extract picture characteristics independently in the case of this sort of neural network. In image classification problems, complex and pre-trained CNNs with millions of parameters are frequently utilized. Their complete training is difficult since it is a time-consuming and labor-intensive procedure^34^. With developments in machine learning (ML) principles, significant gains in agricultural activities have been noticed. The capacity to extract features automatically generates an adaptable nature in deep learning (DL), especially CNNs, which achieves human-level accuracy in a variety of agricultural applications, prominent among which are crop/plant recognition, fruit counting, land cover classification, weed/crop discrimination, and plant disease detection and classification^35^.

### Methods

#### Transfer learning.

Data Transfer Learning (DTL) is a strategy in which knowledge derived from the data is transferred to solve various but associated assignments to train the CNN, including new data that often comes from a lower population^36^. To initialize the models and pre-train two profound convolutionary neuro-network models, transfer learning was used: AlexNet and GoogleNet.

**Figure 1:**
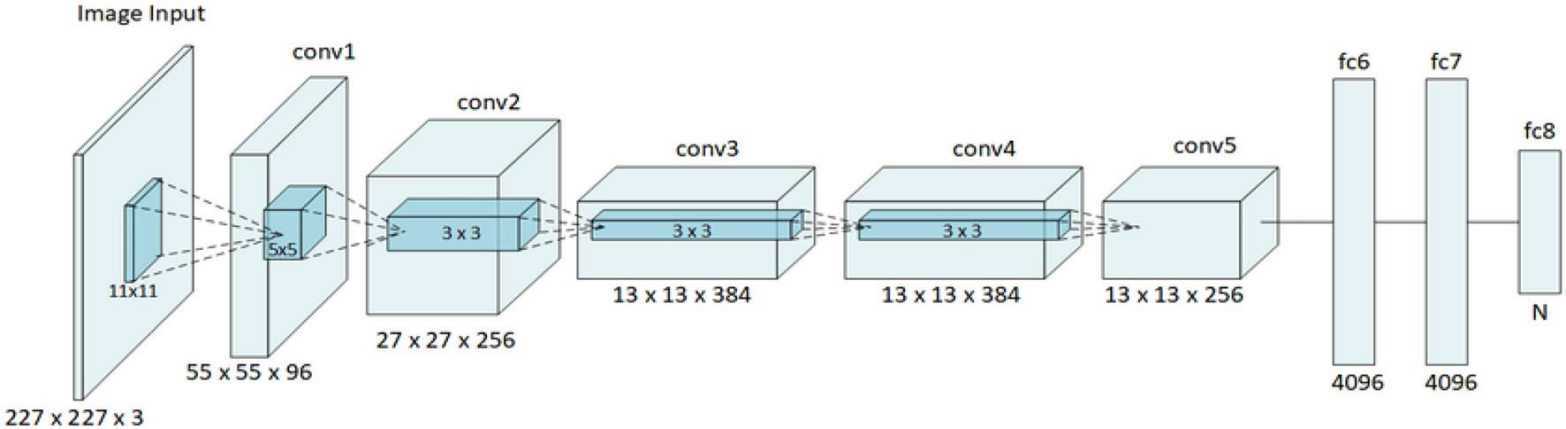
An illustration of AlexNet layers.

AlexNet is expected to be the first recommended deep CNN technology due to its remarkable outcomes for the identification and classification functions on image data^37^. In an attempt to improve hardware constraints and obtain the total functionality of deep CNN, AlexNet was trained on two parallel GPUs. In AlexNet, the CNN depth was widened from only five layers in the LetNet CNN to eight layers in the way to produce CNN appropriate to different data sets of images. Dropout, ReLU, and pre-processing are major attributes to attain significant improvement in computer vision applications. The common 8 layers are five convolutional layers, two fully connected hidden layers, and one fully connected output layer, as shown in Fig. 1^38^.

In this study, we replaced the 1000 classes that the original AlexNet had, with only 2 classes which we evaluated in this paper, healthy images and diseased images of 10 different plants as illustrated in the dataset description.

**Figure 2:**
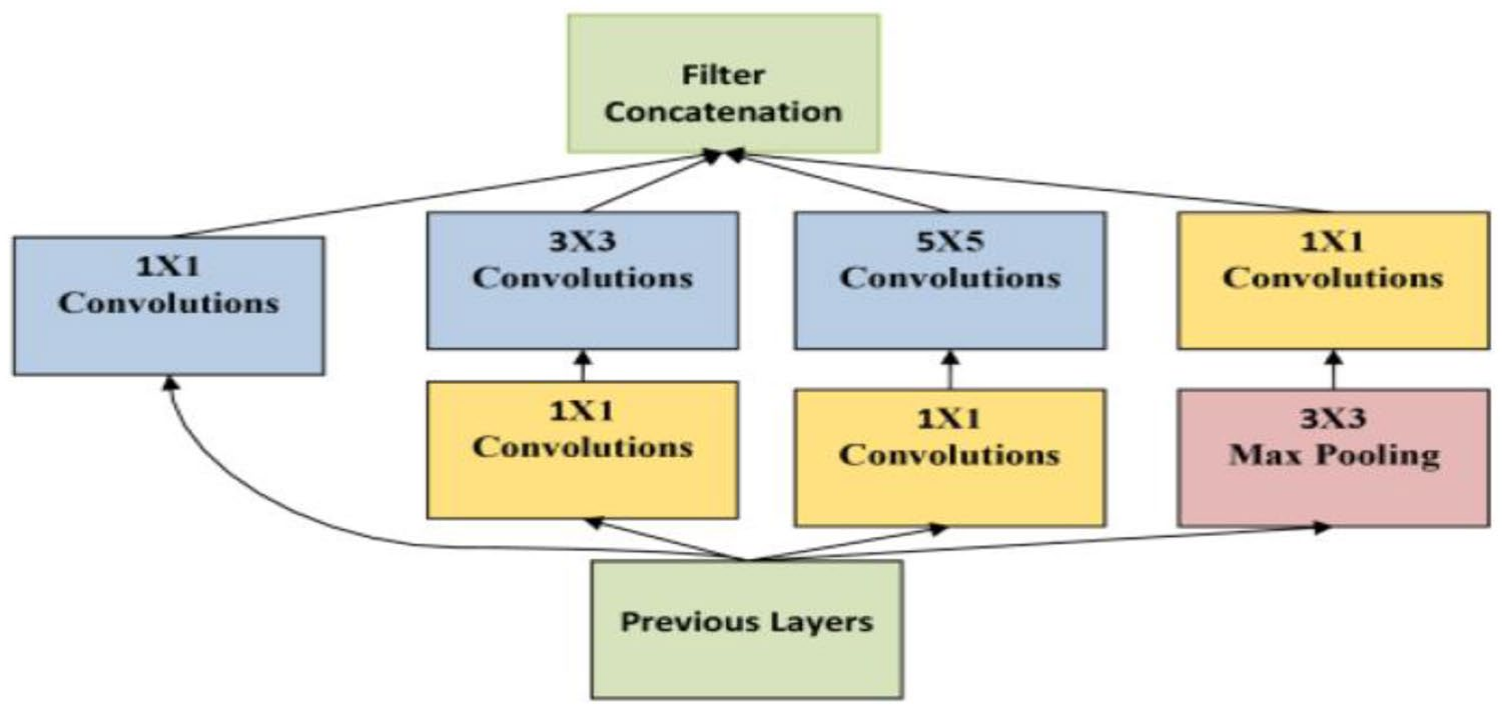
GoogleNet network structure diagram.

GoogleNet consists of 22 layers deep CNN that is a version of the inception network established by Google researchers. The design of the GoogleNet structure resolved many constraints that appeared for large networks, primarily out of the use of the Inception module. The structure diagram of the GoogleNet network is shown in Fig. 2^39^.

GoogleNet consists of inception modules, so its architecture is complex. GoogleNet is looked like one of the initial CNN architectures to resist successively accumulating convolutions and pooling layers. In addition, GoogleNet plays a vital role in consideration of storage and power, since accumulating all tiers and combining different restrictions would take time for computation and will result in higher costs of memory^40^.

#### Support vector machine

The deep feature extraction technique necessitates the training of a classifier method with the extracted features. Vapnik's SVM was utilized as a classifier in this study^41^. It has been found that the SVM classifier outperforms others in several agricultural image categorization tasks.

The support vector machine is a classifier with a linear or non-linear relationships that is capable of distinguishing between two different types of objects. SVMs are machine learning approaches focused on cambered improvement that operate as stated by the concept of structural risk reduction. These approaches are separate of distribution, as it does not need any details on the common distribution functions^42^. SVM training can be illustrated with algorithm 1^43^.
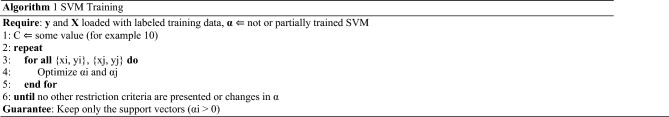


While a hyperplane classifier can distinguish between 2 classes, certain categories surpass the highest distance set as the most effective separation hyperplane. The objective of SVM is to construct an ideal hyperplane space by utilizing training sets^40^.

The main idea behind using SVM to solve a classification issue is to find a hyperplane that best separates data from two groups. The formula for a linear SVM's output is presented in Eq. (1), where $\overrightarrow{w}$ is the hyperplane's normal vector and $\overrightarrow{x}$ is the input vector. Margin maximization may be thought of as an optimization issue: reduce Eq. (2) subject to Eq. (3) where *yi* and $\overrightarrow{x}$ are the SVM's correct output and input vector for the ith training sample, respectively^44^.

1 $$ u{ } = { }\vec{w}.{ }\vec{x} - {\text{b}} $$

2 $$ 1/2\left\| {\vec{w}} \right\|2 $$

3 $$ yi{{(\vec{w}}}{{.\vec{x} - b)}} \ge {1,}\forall i $$

SVM is a binary classifier that can only distinguish between two classes and does not handle multi-class classification issues. One approach to classification of multi-class using SVMs is to build a one-to-one group of classifiers and forecast the class picked by the majority of classifiers^45^. While this allows for the creation of K (K + 1)/2 classifier for the classification issue with K classes, the classifiers training time may be decreased because the training data set for every classifier is lower. In this article, SVM is used to analyze data in addition to CNN techniques such as AlexNet and GoogleNet.

#### Grey wolf optimization (GWO) algorithm

**Figure 3:**
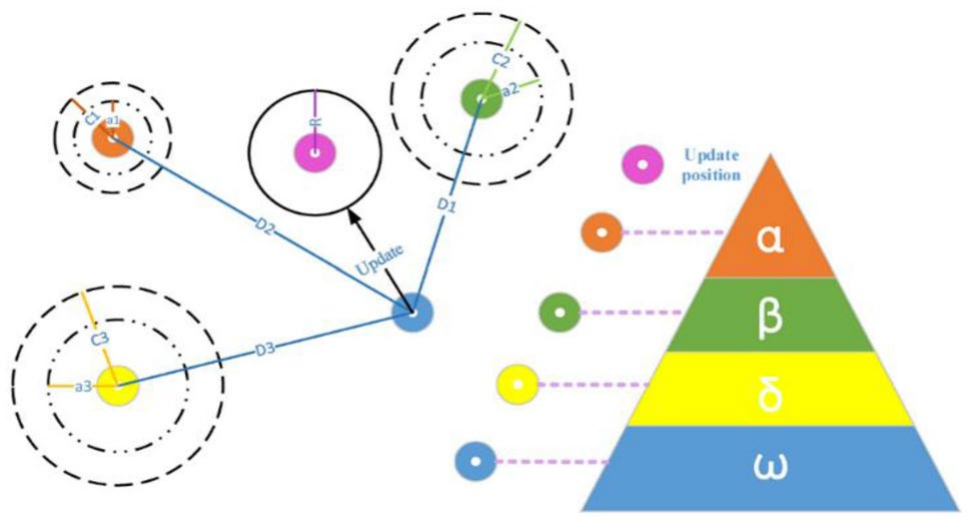
GWO’s social structure and position update method.

Mirjalili et al. proposed the grey wolf optimizer (GWO) as a novel swarm intelligence method^46^. The GWO method has been successfully utilized and applied in a variety of research. The primary inspiration for the GWO algorithm came from the social pursuit of grey wolves in nature. Figure 3 depicts the social hierarchy as well as an instance of the position update process^47^.

In the GWO algorithm, the first, second, and third best-recommended solutions are alpha (α), beta (β), and delta (δ). Omega is projected to be the outstanding solution. The wolves can be presented in a form that is representable mathematically in Eqs. (4–8) during the hunting process:

4 $$\overrightarrow{D}=\left|\overrightarrow{C}. {\overrightarrow{X}}_{P}-{\overrightarrow{X}}_{\left(t\right)}\right|$$

5 $${\overrightarrow{X}}_{\left(t+1\right)}={\overrightarrow{X}}_{P\left(t\right)}-\overrightarrow{A}.\overrightarrow{D}$$

6 $$\overrightarrow{A}=2 \overrightarrow{a}. \overrightarrow{rand1}-\overrightarrow{a}$$

7 $$a=2\times (1-\frac{t}{{t}_{max}})$$

8 $$\overrightarrow{C}=2. \overrightarrow{rand2}$$

where x is the grey wolf's vector position, xp is the prey's vector position, D is the distance between x and xp, t is the current iteration number, and A and C correspond to component-wise multiplication.

The "A' parameter is given in a [− a, a] random value according to the "a" value. Whether a gray wolf attacks or not, the value of A can be determined. As a result of the calculation, the gray wolf is exceptionally close to the hunt and can attack at any time if |A < 1| status is available. The gray wolf leaves a beast in the case of |A > 1|, hoping for a new beast. Another critical parameter of control, C, is recognized as the exploration component of the algorithm and may include random values within the range [0, 2]. This variable leads to a random behavior of the algorithm that prevents an optimization at optimum local values. This condition happens if the random conduct is minimized by |C < 1| or else |C > 1|^47^.

To mimic grey wolf hunting behavior, Eqs. (9–14) show how grey wolves are positions updating of α, β, and δ wolves. It is accepted that the wolves of α, β, and δ are closest to the prey and attract the rest of the wolves to the prey area. The grey wolf population can use the following formulae to determine prey position:

9 $${\overrightarrow{D}}_{\alpha }=\left| {\overrightarrow{C}}_{1} . {\overrightarrow{X}}_{\alpha }-\overrightarrow{X}\right|$$

10 $${\overrightarrow{D}}_{\beta }=\left| {\overrightarrow{C}}_{2} . {\overrightarrow{X}}_{\beta }-\overrightarrow{X}\right|$$

11 $${\overrightarrow{D}}_{\delta }=\left| {\overrightarrow{C}}_{3} . {\overrightarrow{X}}_{\delta }-\overrightarrow{X}\right|$$

12 $${\overrightarrow{X}}_{1}={\overrightarrow{X}}_{\alpha }(t)-{\overrightarrow{A}}_{1}.({\overrightarrow{D}}_{\alpha })$$

13 $${\overrightarrow{X}}_{2}={\overrightarrow{X}}_{\beta }(t)-{\overrightarrow{A}}_{2}.({\overrightarrow{D}}_{\beta })$$

14 $${\overrightarrow{X}}_{3}={\overrightarrow{X}}_{\delta }(t)-{\overrightarrow{A}}_{3}.({\overrightarrow{D}}_{\delta })$$

The locations are determined from Eqs. (12–14) is utilized to modify the next location of the wolves by Eq. (15):

15 $${\overrightarrow{X}}_{\left(t+1\right)}=\frac{\left({\overrightarrow{X}}_{1}+{\overrightarrow{X}}_{2}+{\overrightarrow{X}}_{3}\right)}{3}$$

where xt + 1 is the location of the next iteration. Using Eq. (15) to find a new location for the leading wolves drives the Omega wolves to change their locations to converge with prey.

The GWO algorithm sequence consists of three steps: initialization, fitness calculation, swarm individual position updates, and the best result generation. The optimization process starts with the starting value for all control parameters, and all gray wolves are altered in regular intervals. The fitness function is then calculated based on the initial data, and the best solutions are identified as wolves of alpha, beta, and delta. The next step is to upgrade all gray wolves other than delta, beta, and alpha wolves. The next step is to renew all gray wolves' positions and controller parameter values, followed by Alpha, Beta, and delta wolves. Finally, the alpha wolf returns its optimal position value.

#### Fog computing and IOT

The providers of cloud computing frequently utilize data centers that consider a variety of factors, including energy consumption and user proximity. Thus, the cloud layer, the top layer, often comprises a cloud infrastructure made up of data centers that provide resources and amenities that are dynamically assigned according to the demands of the users. These services could include networking, storage, and server (rendering tools, computational power, and so on) capabilities^48^. Fog Computing attempts to bring processing capabilities closer to end-users, preventing overuse of Cloud resources, further lowering computational burdens, enhancing load balancing, and shortening wait times^49,50^.

**Figure 4:**
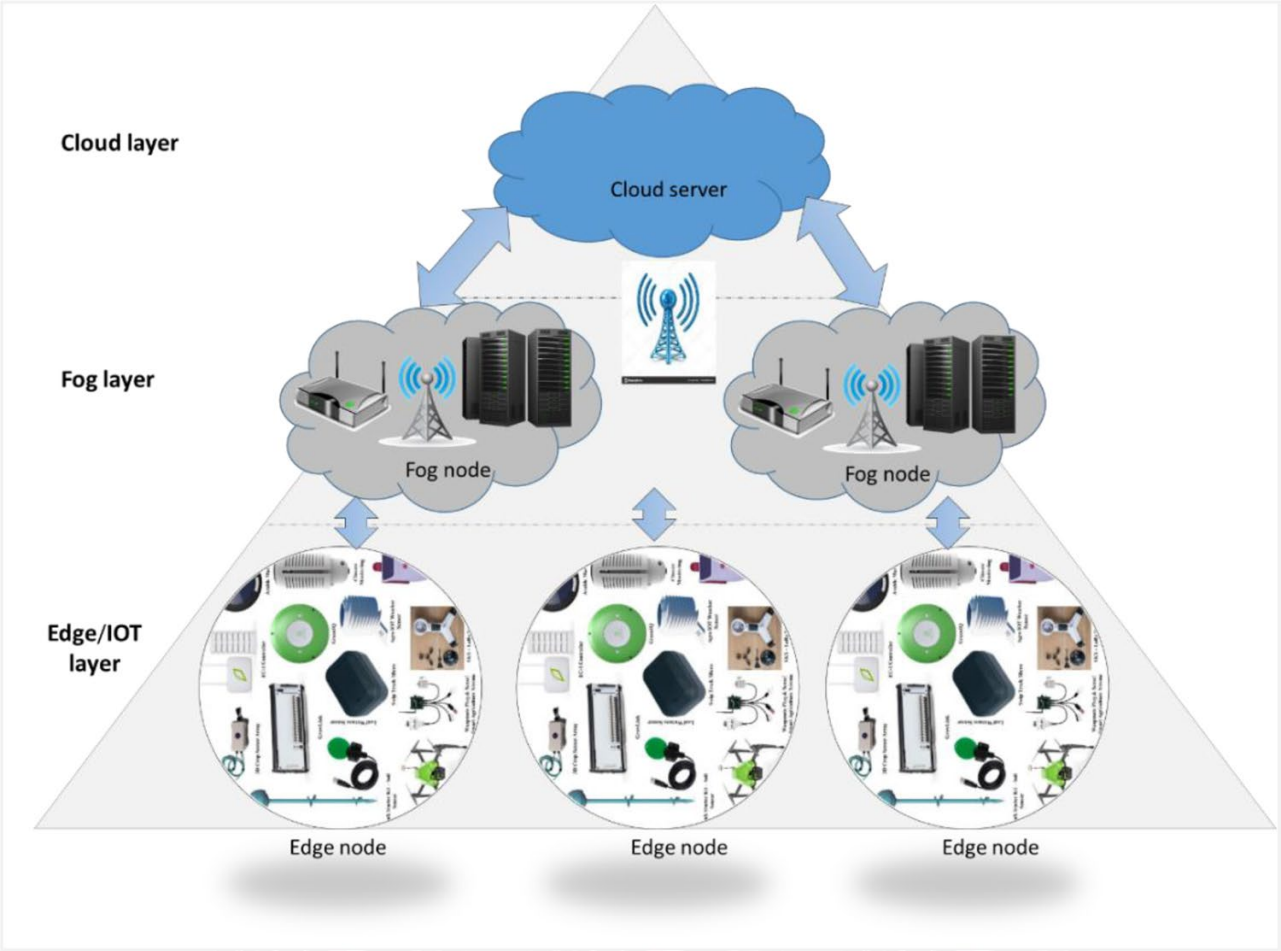
Smart agriculture IoT with edge, fog, and cloud computing.

The Internet of Things (IoT), which represents the future of communications and computers, is a breakthrough technology. IoT is now used in almost every sector, including intelligent cities, intelligent traffic control, and intelligent homes. The deployment of IoT is wide and may be applied in any field. IoT aids in better resource and crop management, crop monitoring, cost-effective agriculture, and increased quantity and quality. Air temperature sensors, soil moisture, soil pH, water volume, humidity, and other IoT sensors are employed^47^. Figure 4 shows IoT in agriculture using edge computing, fog computing, and cloud computing.

The key benefits of IoT in agriculture are discovered in these points^51^:

- Community agriculture in rural and urban regions, utilizing software and hardware resources as well as vast amounts of data.
- Quality and logistical traceability of food security that allows reduced costs via real-time decision-making data.
- Business strategies established in the agricultural setting that enable direct consumer contact.
- Crop surveillance allows cost savings and machine robbery avoidance.
- Systems of automatic irrigation that operate based on soil moisture levels, and temperature measured by sensors.
- Environmental characteristics are automatically collected via sensor networks for subsequent analysis and processing.
- Large quantities of data are analyzed by decision support systems to increase production and operational efficiency.

At the end of this section, we can summarize this paper in 3 folds; the first is applying DL models (AlexNet, GoogleNet) to extract features from plants. Secondly is using an optimization algorithm called the modified grey wolf optimization algorithm for eliminating the redundant features. The third is the classification of the output images using the support vector machine. The above techniques are divided to be used some processes in Fog and some processes in cloud computing. The next section introduces the architecture of the proposed solution using the deep learning techniques referred to above.

## Proposed methodology

As technology advances, smart agricultural solutions are becoming more prevalent. Since then, technology has returned to agriculture with the latest trends and techniques it has produced. A significant advantage of smart agriculture is connecting to existing 3G and 4G networks using existing hardware and software. For smart agriculture solutions, it speeds up setting up hardware, resulting in the various successful implementation of IoT in agriculture that can run in a fog or cloud environment. There will be an evolution from the existing standard mobile computing scenario of smartphones and their apps to the connection of gadgets around us to help solve a real-world problem^52^. We’ll discuss in this section the proposed methodology based on the mentioned transfer learning, pretraining methods, and the optimization algorithm on fog and cloud computing using IoT sensors common in the problem statement of this paper.

**Figure 5:**
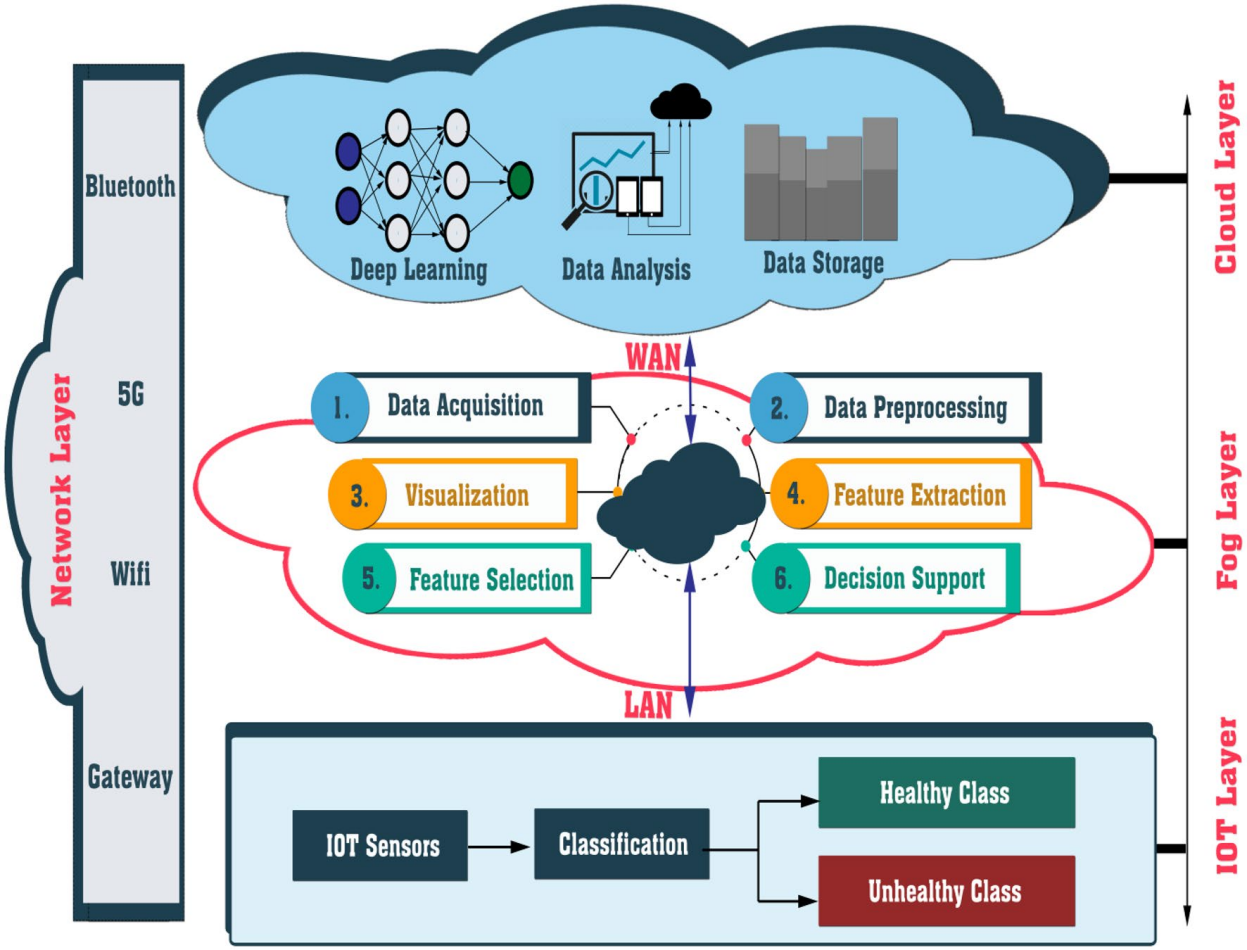
Block diagram of the proposed IoT smart agriculture network architecture.

Figure 5 shows the block diagram of the proposed IoT smart agriculture network architecture which consists of three layers. The first layer contains the IOT devices that are used for different purposes in agriculture. Many technologies are being used in IoT agricultural solutions which have an important role in modernizing the services of IoT agriculture. Examples of these technologies are cloud and edge computing, machine learning and big data analytics, communication networks and protocols, and robotics. The second layer presents the sequence of work in this paper from collecting the images from IoT sensors then preprocessing these images if they need to resize, or normalize, or removing noise according to the recommended DL algorithms in this paper (CNN, SVM). All processes happened on the images from collecting it till detection of plant diseases are applied on fog environment to facilitate the function of scalability and stability that are advantages of fog computing. The third layer is connecting with cloud computing for henting resources for further and large processing. The other proposed models don’t suitable for cloud or fog computing, so we proposed a new model for plant disease detection using machine learning techniques by the Internet of Things (IoT) sensors that can run on fog or cloud environments.

The proposed model depends on deep learning, transfer learning, and shallow machine learning. In deep learning, multi-hidden layers are stacked for learning objects significantly. These layers require a training process including “fine-tuning” for adjusting the weights slightly of DNN discovered during the procedure of backpropagation. In turn, following an efficient training procedure, DL nets can categorize, extract characteristics, and give a decision effectively and accurately. In the proposed model, we use transfer learning to optimize different pre-innovated CNNs architectures to the datasets.

**Figure 6:**
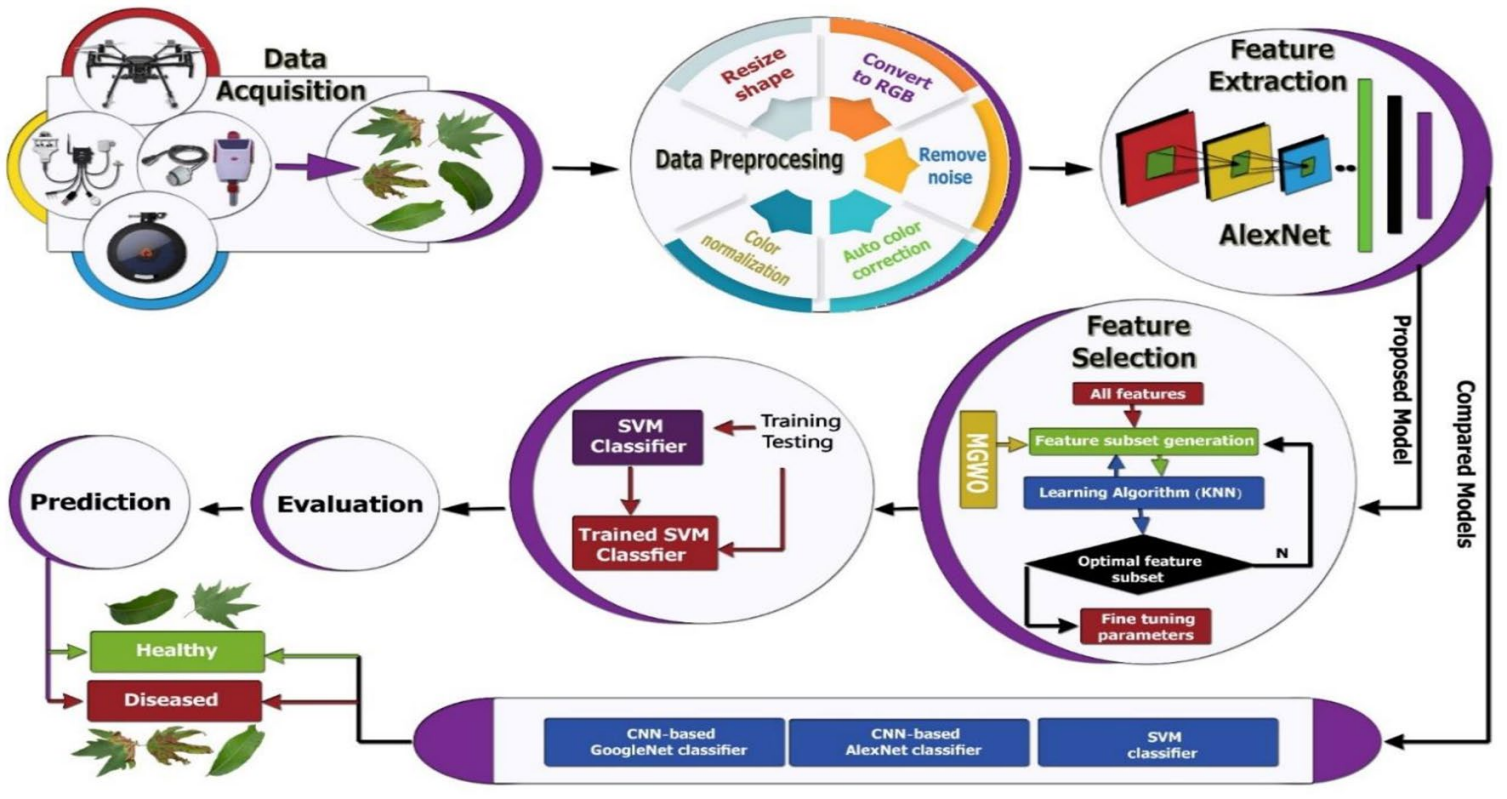
Dataflow diagram of the proposed methodology.

As seen in Fig. 6, the proposed model starts by a data acquisition layer in which images are collected for different plants. This acquisition procedure was entirely wi-fi enabled, which means that the camera and the computer were linked with each other via the internet. In the preprocessing phase, the images are reconstructed and resized since the images are taken from various sources and their dimensions vary. In addition, the model layer of each of these products needs separate image dimensions to be managed. Therefore, the input image size is adjusted to fit the templates that are used in this analysis.

The feature extraction layer comes after image enhancements that represent the layer in which most of the calculations are carried out. The calculations include extracting image data set features and preserve the spatial relationship between image pixels. A pre-trained CNN, AlexNet, was used as feature extraction and we extracted the ’fc7’ layer as our feature extracting layer. After extracting the features, it is the role of feature subset selection to reduce the features and eliminate the irrelevant features. The proposed model makes use of a modified version of the grey wolf optimization algorithm. The details of the modified grey wolf optimization algorithm (MGWO) are explained in the next subsection. After that, the generated features set were utilized to train the SVM. Once we get the baseline SVM, we use a validated data set to adjust SVM parameters.

### Modified grey wolf optimization algorithm (MGWO)

Mirjalili showed that The GWO algorithm tends to become stuck at optimal local values because of the small number of control parameters utilized in its simplest form. Because of this, researchers modified GWO by adding additional controls and changing control parameter values. According to their findings, the alpha-wolf was more powerful than the delta—and beta-wolf when searching for food. So, it's possible to acquire better outcomes in tests in this manner. For this reason, there is much research in the literature that has adapted and developed the grey wolf algorithm in various sectors. As a result of this, it produces superior outcomes in tests^47^.

The parameter adjusted equation for the "a" parameter was used in this study to improve the method^50^ significantly. However, instead of using the usual GWO Eq. (7), this study uses Eq. (16) to derive the parameter "a" instead.

16 $$a=2\times ({e }^{\frac{-t\times s}{tmax}})$$

"s" is only added in Eq. (16) to "a", and it reflects the total number of individuals in the swarm, as seen in Eq. (16). Standard GWO has a linearly decreasing "a" parameter, which prevents the algorithm from settling on local minimum values. Researchers found that as the "a" attribute approaches 0, it not only keeps the algorithm from reaching a locally minimal value but also considerably enhances its strength. Therefore, the method converges on the optimal values faster when this parameter is reduced from 2 to 0. So, the program has sped up and parabolically slowed down from 2 to 0.

Moreover, it can be seen from the governing Eq. (15) that the dominants perform a similar function in the searching procedure; each of the grey wolves converges or flees away from the surroundings with an average weight of the beta, delta, and alpha. Even if the alpha is closest to the victim at first, it may be distant from the eventual result. Only the alpha position should be considered in Eq. (15) at the beginning of the search operation, or its weight should be substantially more significant than that of other dominants. Equation (15)'s average weight, on the other hand, contradicts the grey wolf social hierarchy idea. If the pack's social hierarchy is strictly observed, the alpha is in charge, which could mean that he/she will always be the closest to the prey. This indicates that the alpha wolf's weight in Eq. (15) should never be smaller than that of the delta and beta wolves. As a result, the beta's weight should always be more than the delta's. In light of these concerns, the authors^53^ further hypothesize the following:

1. The dominants surround a supposed prey when it is being searched for, but they do not surround an actual prey when being hunted. As their social hierarchy dictates, the dominant grey wolves encircle the prey in order of their dominance. The alpha is the closest wolf in the pack, followed by the beta and the delta. Omega wolves play a role in this process, passing on their superior positions to the dominants.
2. Alpha controls the search and hunting process, while beta has a minor role, and delta has an even smaller one. A wolf's position changes if an alpha wins out over his/her peers.

Equation (15) should not use the same updating procedure for the positions as the previous hypotheses. Thus, the alpha weight should be near 1.0 at the beginning, whereas delta and beta weights could be close to 0. According to Eq. (15), the delta, beta, and alpha wolves should surround the victim at the final stage. During the entire process of searching, the alpha is always found by the beta, and the beta always finds the delta because he/she is always ranked third. As a result, the beta and delta weights are determined by the total number of iterations. Alpha's weight should be reduced, and beta and delta's weights should rise.

In mathematics, the above ideas could be stated. When adding up the weights, ensure that they're all varying and that the aggregate is capped at 1.0. As a result, Eq. (15) is altered to the following:

17 $${\overrightarrow{X}}_{\left(t+1\right)}={w}_{1}{\overrightarrow{X}}_{1}+{w}_{2}{\overrightarrow{X}}_{2}+{w}_{3}{\overrightarrow{X}}_{3}$$

$${w}_{1}+{w}_{2}+{w}_{3}=1$$

As a second rule, when calculating the alpha w1, beta w2, and delta w3 weights, they should always satisfy w1 >  > w2 > w3. Along with the search technique, the weight of the alpha would be adjusted from 1.0 to 1/3. While doing so, we'll boost beta and delta's weights, increasing them from 0.0 to 1/3 in the process. W1 can be described using a cosine function if we limit the angle range to be between [0, arccos (1/3)]. The weights should be adjusted based on the total iterations or "it" as a third point. And we recognize that w2⋯w3 ⟶ 0 if it = 0 and w1, w2, w3 ⟶ 1/3 when it ⟶ ∞. As a result, we present an arc-tangent function that changes from 0.0 to π/2. And then, somehow, cos (π/4) = sin (π/4) = − 2 √ /2, so different angular parameter φ was organized as seen below^53^:

18 $$\mathrm{\varphi }=\frac{1}{2}\mathrm{ arctan}(it)$$

Given that w2 would be maximized from 0.0 to 1/3 alongside it, we assume that it includes cos φ and sin θ and θ ⟶ arccos (1/3) when it ⟶ ∞; hence,

19 $$\uptheta =\frac{2}{\uppi }\mathrm{arccos}\frac{1}{3}.\mathrm{arctan}(it)$$

when it ⟶ ∞, θ ⟶ arccos (1/3), w2 = 1/3, we can then formulate w2 in detail. The following is a new updating method for positions with variable weights that are based on these principles:

20 $${w}_{1}=\mathrm{cos\theta },$$

21 $${w}_{2}=\frac{1}{2}\mathrm{sin\theta }.\mathrm{ cos \varphi },$$

22 $${w}_{3}=1-{w}_{1}-{w}_{2}$$

**Figure 7:**
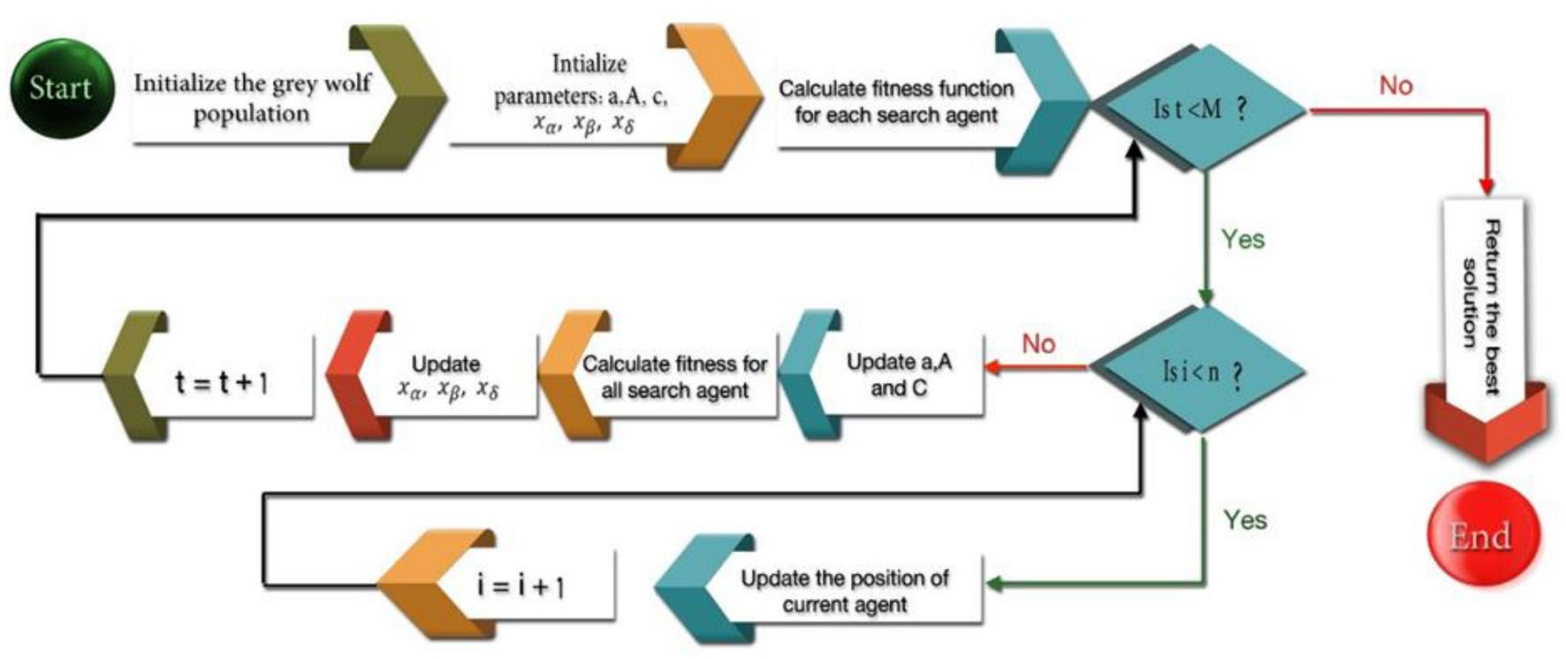
Flowchart of the grey wolf optimization algorithm.

The flowchart of the Modified Gray Wolf Optimization (MGWO) algorithm is shown in Fig. 7.

The pseudocode of the MGWO is presented in algorithm 2.
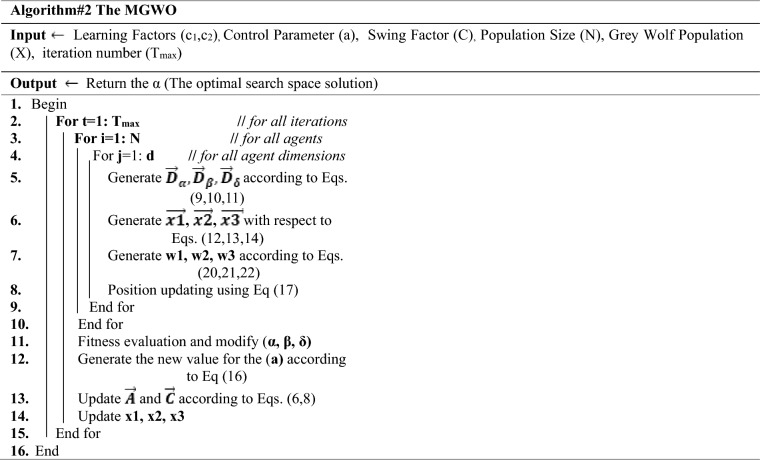


## Experimental results and discussion

### Performance measures

True positives, true negatives, false negatives, and false positives, and are displayed separately in the table, in two rows and two columns, accordingly (sometimes also referred to as a confusion matrix). In this way, the classification proportion can be studied in greater detail (accuracy). An unbalanced data collection (i.e., when the number of observations in different classes changes dramatically) will lead to inaccurate conclusions. Sensitivity and specificity are also valuable traits to have. As shown in Table 2^54^, the most widely used measures are used to create the confusion matrix (Data science, 2019). Five measurements are utilized in this article to gauge our work performance, these measurements are shown in Table 3.

**Table 2:** The confusion matrix.

Actual output		Output class	
		Positive	Negative
Real class	Positive	True positives (TP)	False negatives (FN)
	Negative	False positives (FP)	True negatives (TN)

**Table 3:** Performance measures.

Metric	Meaning	Calculation	Equation
Sensitivity	(True Positive Rate) assesses correctly detected proportions of positive (i.e., the balance of those correctly detected as having a condition)	Sensitivity (Recall) = $\frac{\mathrm{TP}}{\mathrm{TP}+\mathrm{FN}}$	(23)
Specificity	(The fraction of the negative that is correctly detected (i.e. the ratio of the non-conditionality (uninfluenced) erroneously identified as non-condition) (True Negative Rate)	Specificity = $\frac{\mathrm{TN}}{\mathrm{TN}+\mathrm{FP}}$	(24)
Precision	The ratio for all positive examples adequately classified, and the total number of positive models forecast. In positive prediction, it shows the correctness achieved	Precision = $\frac{\mathrm{TP}}{\mathrm{TP}+\mathrm{FP}}$	(25)
Accuracy	The ratio of the entire number of correct forecasts. Precision is the proximity between the measures to a given value in a set of measurements, while precision is the proximity	Accuracy = $\frac{\mathrm{TP}+\mathrm{TN}}{\mathrm{TP}+\mathrm{TN}+\mathrm{FP}+\mathrm{FN}}$	(26)
F1 score	The weighted (sensitivity) and the accurate average of recall. F1, if you are trying to balance precision and reminder, maybe the right choice	$\mathrm{F}1\mathrm{ Score}=2\times \frac{\mathrm{Precision}\times \mathrm{Recall}}{\mathrm{Precision}+\mathrm{Recall}}$	(27)

### Experiment 1

We provided the results as two experiments**.** For the first experiment, a modified grey wolf optimization method (MGWO) for feature selection is being evaluated. When developing a machine-learning model, feature selection is becoming increasingly important. The feature selection process involves deleting irrelevant or redundant characteristics and picking the ideal subset of features that better categorize patterns that belong to different plants. The evaluation is made by using fifteen standard feature selection datasets. The overall properties of these datasets are given in Table 4^55^.

**Table 4:** Datasets used for evaluating the MGWO.

ID	Dataset	No. of instances	No. of attributes	No. of classes
1	Breast cancer	699	9	2
2	Climate	540	20	2
3	Diabetic	1151	19	2
4	Ionosphere	351	34	2
5	kc1	2110	21	2
6	Lung Cancer	226	23	2
7	lymphography	148	18	4
8	pc1	1109	21	2
9	Stock	950	9	2
10	Segment	2310	19	7
11	WineEW	178	13	3
12	Tic-tac-toe	958	9	2
13	Vote	300	16	2
14	WDBC	569	30	2
15	Zoo	101	16	7

Using a random seeding strategy, a random population of n wolves or search agents is formed in the first part of the procedure. An ideal solution is found when the number of features "d" equals that of the original dataset features set. When selecting features for purity classification, make sure they enhance accuracy. Identify the appropriate characteristics (one value) and discard the rest (zero). Initially, the binary values (0 and 1) were set in each solution.

**Figure 8:**
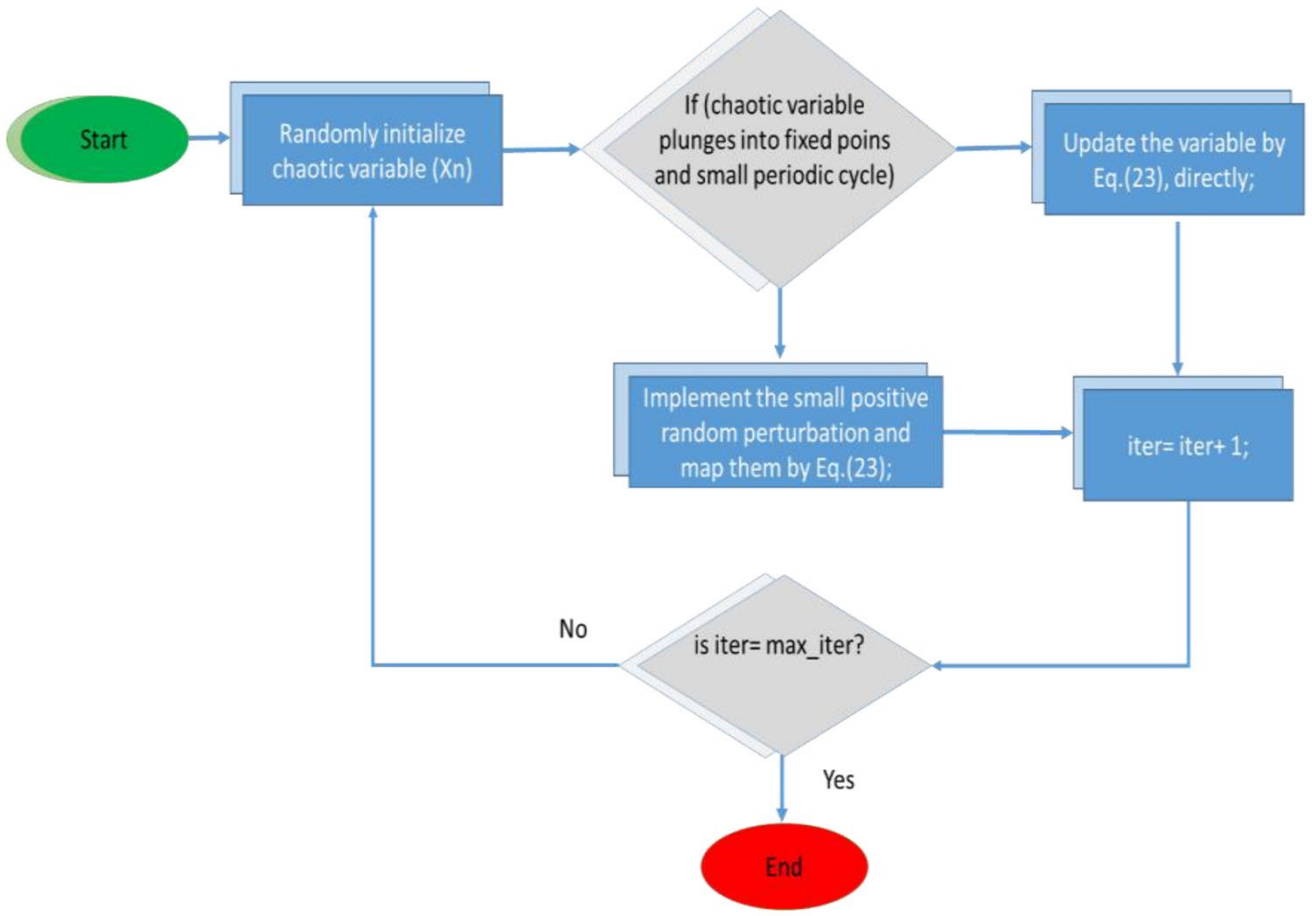
Flowchart of logistic map for initialization.

A large part of GWO's success depends on the development of initial populations. We use chaotic initialization of maps to increase the global convergence speed of the MGWO optimization process. Instead of a standard map, a chaotic map serves to improve the balance of search-and-exploitation skills. The logistics map is one of the most effective chaos-based approaches. It is represented as follows in Eq. (28) ^56^.

28 $$ {\text{X}}_{{{\text{n}} + {1}}} = \, \phi \left( {{\text{X}}_{{\text{n}}} , \, \mu } \right) = \, \mu \times {\text{ X}}_{{\text{n}}} \left( {{1} - {\text{ X}}_{{\text{n}}} } \right) $$

where μ is set to 4, the bifurcation coefficient is also defined. x_n_ means the n_th_ chaotic variable, in other words, x_n_ ∈ (0, 1) in favor of limitations that the initial x_0_ ∈ (0, 1) of severely static periods (0, 0.25, 0.5, 0.75,1). Figure 8 of the logistic map shows a consistently divided sequence, which prevents it from effectively immersing into minor regular cycles.

Because the problem has more than one objective, it is understood to be a multi-objective problem^57^. Following steps must be taken to solve the multi-objective issue of selecting optimal features. The first is to produce the highest accuracy rate, and the second is to eliminate the features to the lowest range. Taking this into consideration, the fitness function of the resulting solution evaluation is configured to balance the aims as follows:

29 $$\mathrm{fitness}=\mathrm{\alpha }{\upgamma }_{\mathrm{R}}\left(\mathrm{D}\right)+\upbeta \frac{\left|\mathrm{S}\right|}{\left|\mathrm{D}\right|}$$

Given that $\left|S\right|$ for the length of the selected subset feature cardinality, $\alpha$ and $\beta$ are generated as parameters for expressing a weight for the percentage of classification accuracy and the total number of selected features respectively, α ϵ [0,1] and β = 1 − α and have been selected concerning the evaluation function, ${\gamma }_{R}\left(D\right)$ denotes the classification error rate. $\left|D\right|$ represents dataset cardinality. So, to find the K neighbors for the KNN classifier, the Euclidean distance^58^ must be calculated as follows:

30 $${\mathrm{EUC}}_{\mathrm{d}}\left(\mathrm{P},\mathrm{Q}\right)=\sqrt{\sum_{\mathrm{i}=1}^{\mathrm{d}}{\left({\mathrm{Q}}_{\mathrm{i}}-{\mathrm{P}}_{\mathrm{i}}\right)}^{2}}$$

Qi and Pi relate to a specific feature in the sample, "i" refer to the number of features in the sample, and d refers to the overall number of features used in the analysis. To reduce overfitting, cross-validation is a popular strategy. Cross-validation with K = 10 is utilized in this paper.

In contrast to binary values, continuous values represent the positions of the search agents formed by the algorithm. A straight application to our situation would be impossible because it is incompatible with the standard binary format for feature selections. Features are selected depending on the problem of feature selection to increase the performance and accuracy of the classification system (0 or 1 in the case of binary features). A transformation function is used to convert a binary search space. The following equations can convert any continuous value into binary with the sigmoid function^57^:

31 $$ {\text{x}}_{{{\text{s}}_{{\text{i}}} }} = \frac{1}{{1 + {\text{e}}^{{ - 10({\text{x}}_{{\text{i}}} - 0.5)}} }} $$

32 $$ {\text{x}}_{{{\text{binary}}}} = \left\{ {\begin{array}{*{20}c} 0 & {{\text{if}}\quad {\text{R < x}}_{{{\text{s}}_{{\text{i}}} }} } \\ 1 & {{\text{if}}\quad {\text{R}} \ge {\text{x}}_{{{\text{s}}_{{\text{i}}} }} } \\ \end{array} } \right. $$

where i = 1, …, d, and ${x}_{binary}$ parameter identified as 0 or 1 by randomly selected value in range: R ϵ [0,1] value compared to ${x}_{{s}_{i}}$, the value of the parameter ${x}_{{s}_{i}}$ which defined in the S-shaped search agent is the value identified by the algorithm calculations (continuous), All trials were conducted on a Windows 10 Pro 64-bit operating system with a Core(TM) i7-8550U CPU running at 1.80 GHz and 1.99 GHz, respectively. To implement the algorithms, we use MATLAB (2018a).

The selected values of the algorithms to be its parameters were collected from the literature to make sure that the algorithms are compared on an equal basis^59^. Although the KNN classification unit for feature selection is a frequent wrapper, it can also be thought of as a learning algorithm that is monitored and characterized by simple and quick learning. There are twenty different runs for each algorithm with a random seed. The maximum number of iterations for all subsequent experiments using the standard k-fold cross-validation is 20.

Multiple observational experiments were conducted on a variety of datasets to determine the best literature values for α and β. Therefore, it has the value of 0.9 for α and has the value of 0.1 for β. The parameters settings of our experiments are shown in Table 5.

**Table 5:** Parameters settings.

Parameters	Value	Parameters	Value
Independent runs	20	A	[0,2]
σ	0.9	R	[0,1]
β	0.1	Dataset dimension	d
K-folds cross-validation	10	Iterations	20
K-neighbors	5	Search agents (n)	5

**Figure 9:**
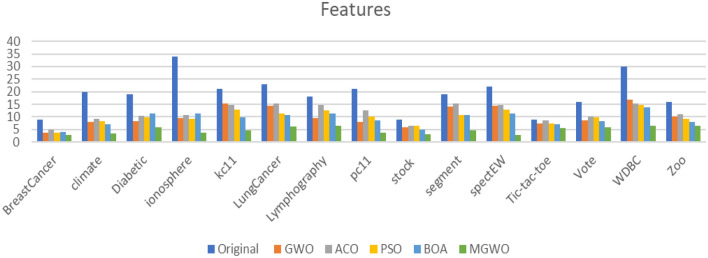
The features reduction for different algorithms.

**Figure 10:**
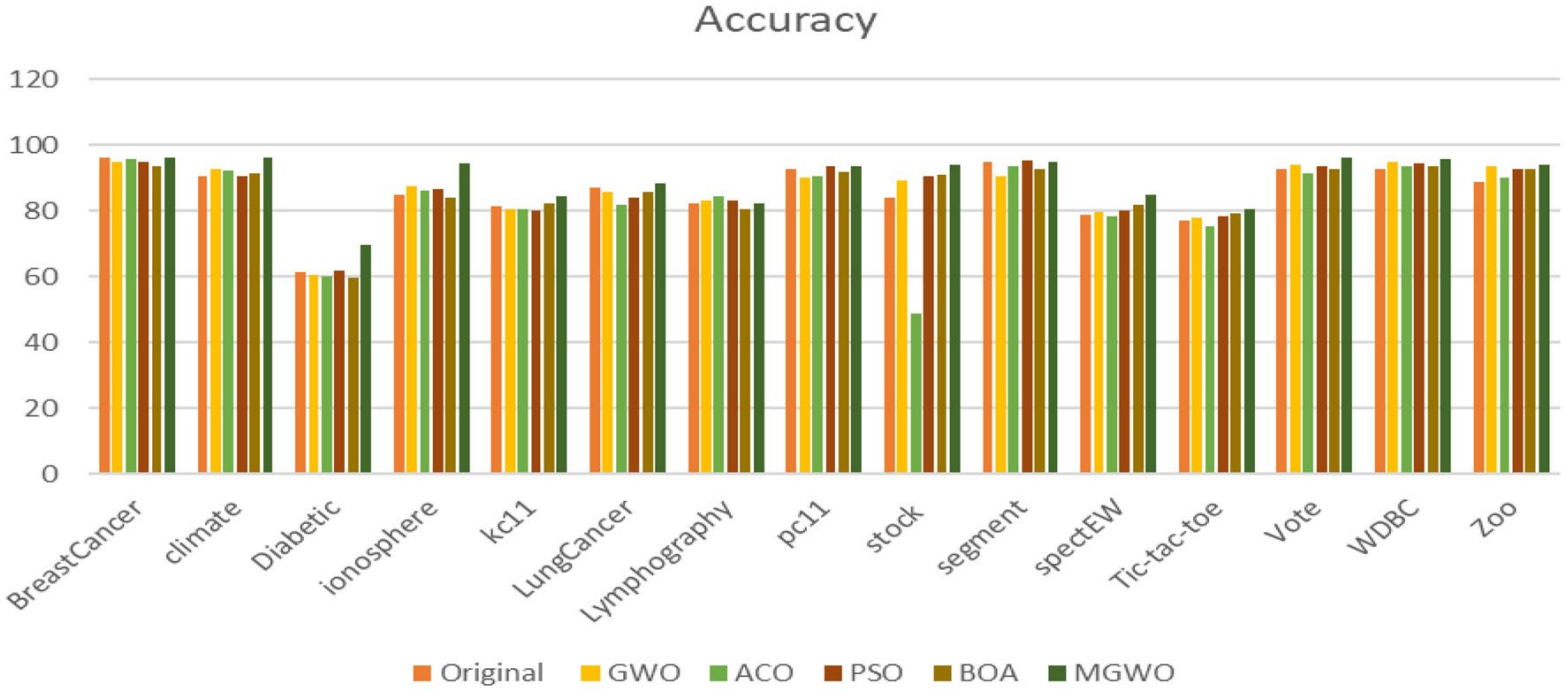
The classification accuracy for different algorithm.

Tables 6 and 7 show the resulted feature and the accuracy respectively. The experimental results are conducted for the standard grey wolf optimization (GWO), the Ant Colony Optimization (ACO), the Butterfly Optimization Algorithm (BOA), the Particle Swarm Optimization (PSO), and the Modified Grey Wolf Optimization (MGWO) algorithms. The experimental results show the superiority of the proposed MGWO in both achieving the least set of features in all the datasets while producing a fair accuracy in most of the utilized datasets. These results are graphically displayed in Figs. 9 and 10.

**Table 6:** The features reduction for different algorithms.

Dataset	Original	GWO	ACO	PSO	BOA	MGWO
Breast cancer	9	3.7	4.8	3.8	3.9	**2.7**
Climate	20	7.8	9.2	8.4	7.1	**3.5**
Diabetic	19	8.2	10.5	9.7	11.2	**5.7**
Ionosphere	34	9.4	10.7	9.11	11.2	**3.8**
kc11	21	15.2	14.8	12.7	9.8	**4.6**
Lung cancer	23	14.5	15.2	11.2	10.7	**6.1**
Lymphographic	18	9.5	14.7	12.4	11.2	**6.4**
pc11	21	7.9	12.4	10.1	8.6	**3.8**
Stock	9	5.8	6.4	6.5	4.9	**3.1**
Segment	19	14.2	15.2	10.8	10.7	**4.7**
spectEW	22	14.5	14.7	12.8	11.4	**2.8**
Tic-tac-toe	9	7.4	8.5	7.4	6.9	**5.5**
Vote	16	8.7	10.1	9.7	8.2	**5.7**
WDBC	30	16.8	15.4	14.6	13.8	**6.4**
Zoo	16	10.2	10.9	9.1	7.8	**6.3**

Significant values are in bold.

**Table 7:** The classification accuracy for different algorithms.

Dataset	Original	GWO	ACO	PSO	BOA	MGWO
Breast cancer	96	95.1	95.55	94.9	93.4	**96.2**
Climate	90.48	92.8	92.1	90.7	91.4	**96.4**
Diabetic	61.6	60.7	60.2	61.7	59.8	**69.7**
Ionosphere	85.1	87.5	86.4	86.5	84.12	**94.5**
kc11	81.4	80.4	80.7	79.9	82.4	**84.4**
Lung cancer	87.1	85.9	81.7	84.12	85.8	**88.2**
Lymphography	82.4	83.2	**84.5**	83.1	80.4	82.4
pc11	92.5	90.18	90.4	**93.5**	91.7	93.4
Stock	84	89.18	48.7	90.7	90.8	**94.13**
Segment	95	90.7	93.4	**95.4**	92.7	94.9
spectEW	79	79.5	78.4	80.14	81.7	**84.9**
Tic-tac-toe	77	77.8	75.2	78.4	79.1	**80.4**
Vote	92.5	94.15	91.5	93.4	92.7	**96.2**
WDBC	92.5	95.1	93.7	94.5	93.5	**95.8**
Zoo	89	93.4	90.2	92.9	92.7	**94.2**

Significant values are in bold.

According to the conclusion of these results, we can say that the MGWO can be used for our plant disease problem.

### Experiment 2

According to the experimental result in the first experiment, the modified grey wolf optimization algorithm (MGWO) can be effective as a wrapper feature selection algorithm. In experiment 2, the core problem of the plant disease classification and prediction is introduced. As discussed in the previous section, the first stage of the proposed model is the feature extraction process where the pre-trained AlexNet CNN is used. This process is performed for ten datasets. The second stage is the feature selection process, in which the MGWO is used as the wrapper feature section method. Lastly, the generated reduced features set were used SVM training. The datasets’ details are discussed in the next subsection.

#### Datasets description

Plants play a crucial role in climate regulation and erosion reduction. To preserve the environment, ecosystem, and living beings, they are both equally necessary to consider. Deciduous and coniferous trees are the most common types. Compared to conifers, deciduous trees have broader and bigger leaves. During the fall, their leaves fall off. This is due to the giant leaf, which allows for more photosynthesis to occur. Trees of this type are famous for their high wood production. There is a coniferous tree or evergreen tree green throughout the year. Leaves have a triangular form and grow upwards in most cases. Even though they have softer wood, they are pretty durable and resistant to various weather conditions^60^.

The data on https://data.mendeley.com/datasets/hb74ynkjcn/1 focuses on plants that contribute both ecologically and economically. As a result, ten different plants, such as Jamun, Lemon, Sukh Chain, Arjun, Pomegranate, Jatropha, Mango, Saptaparni, Guava, and Chinar, have been selected, as shown in Table 8. Images have been divided into two categories: healthy and diseased images. Table 9 shows the dataset description.

**Table 8:** Sample of healthy and diseased leaf images of the plant’s disease dataset.

Data	Healthy	Diseased
P0		
P3		
P6		
P7		
P10		
P11

**Table 9:** Plants diseases datasets description.

ID	Code	Name	Full name	Name of the disease	No. of healthy images	No. of diseased images
1	P0	Mango	Mangifera indica	Anthracnose	170	265
2	P1	Arjun	Terminalia Arjuna	Leaf Spot	220	232
3	P2	Alstonia	Alstonia Scholaris	Foliar Galls	179	254
4	P3	Gauva	Psidium guajava	Fungal disease	277	142
5	P5	Jamun	Syzgium cumini	Fungal disease	279	345
6	P6	Jatropha	Jatropha curcas L	Leaf spot	133	124
7	P7	Sukh chain	Pongamia Pinnata	Chlorotic lesions	322	276
8	P9	Pomegranate	Punica granatum L	Cercospora spot	287	272
9	P10	Lemon	Citrus limon	Citrus Canker	159	77
10	P11	Chinar	Platanus orientalis	Leaf spot	103	120

#### Results

**Figure 11:**
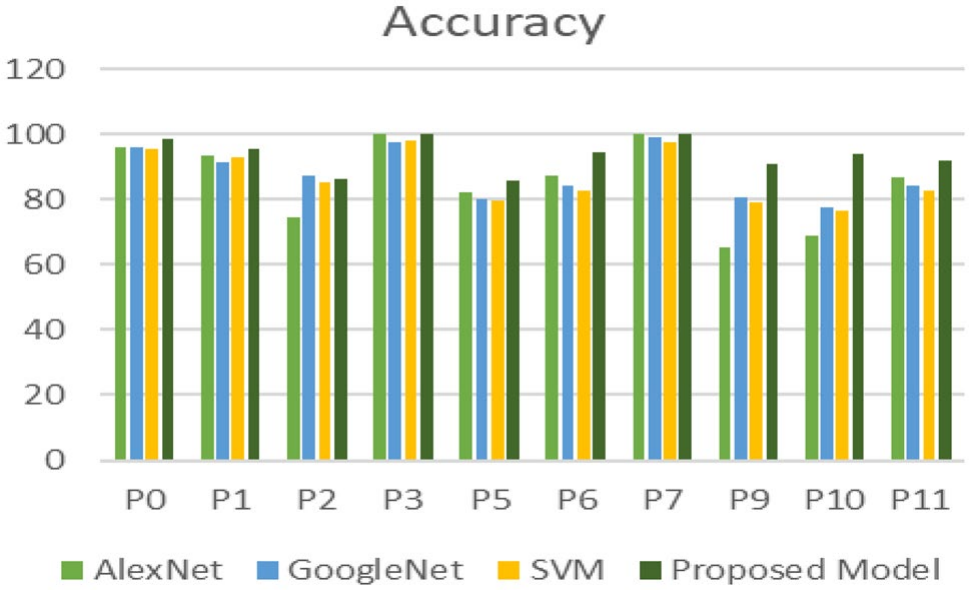
Classification accuracy for the four models.

**Figure 12:**
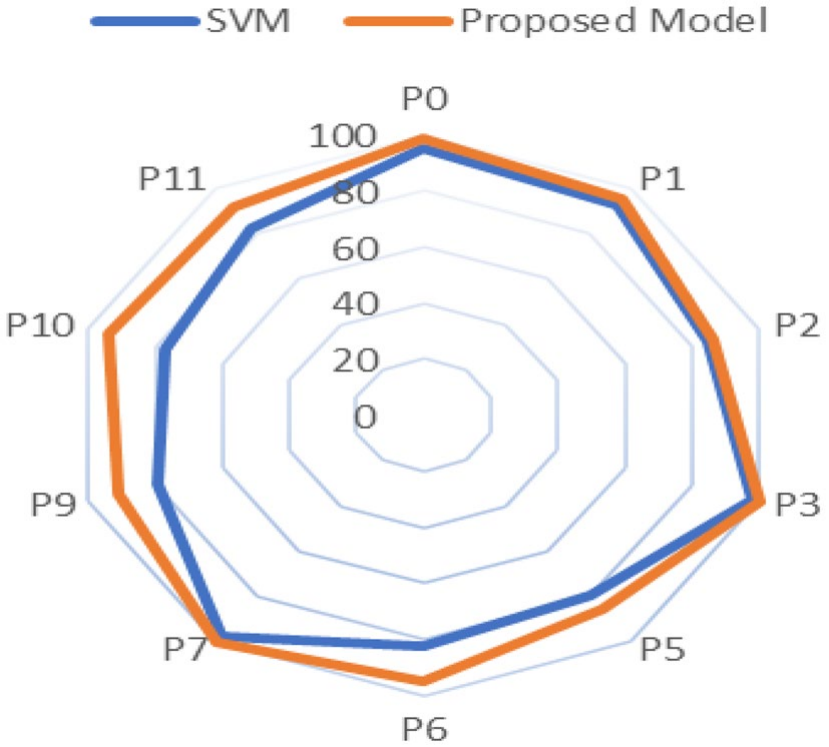
Classification accuracy of the standard SVM Vs the proposed model.

**Figure 13:**
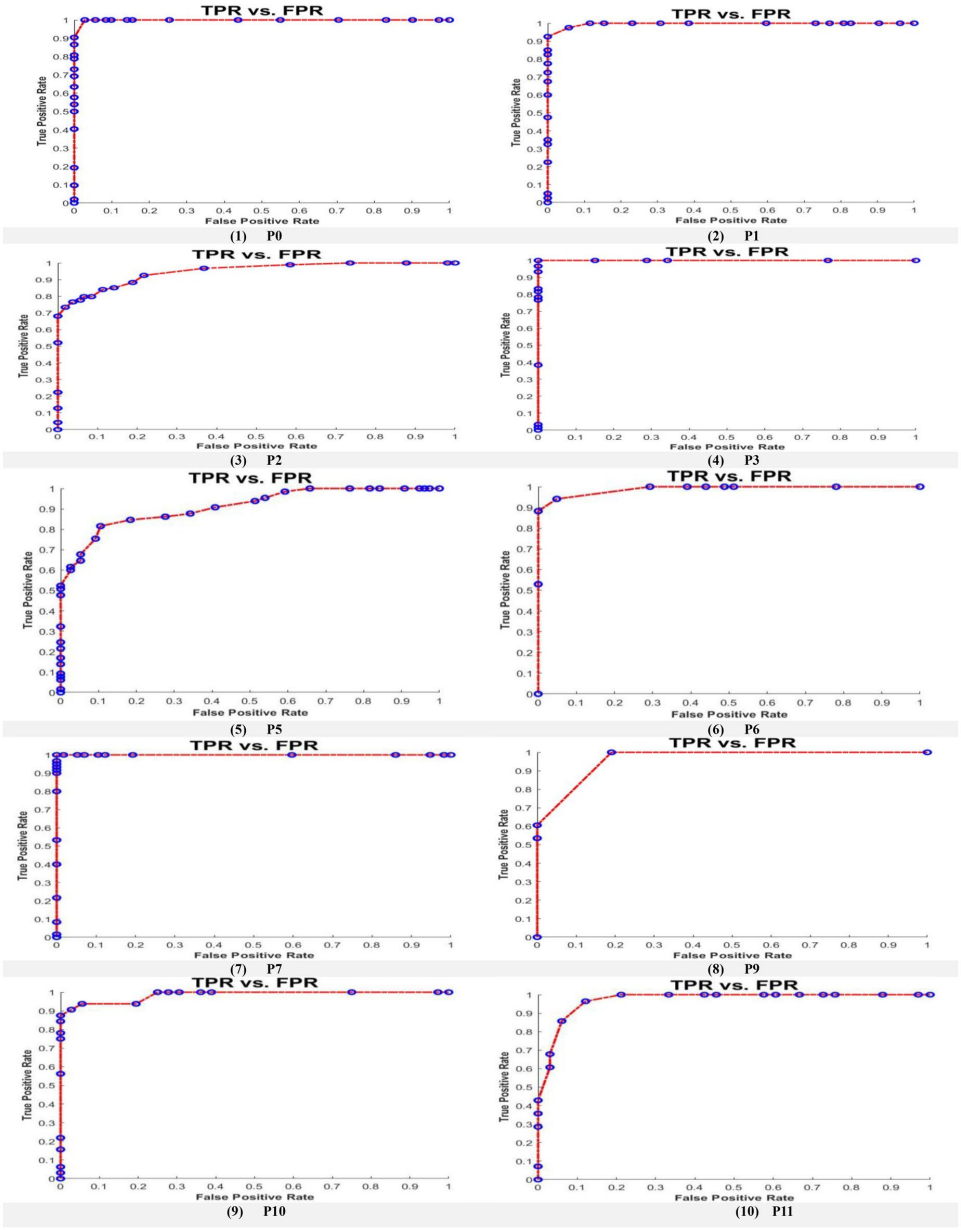
The ROC curve on the test set for the proposed model SVM.

The proposed model (AlexNet as feature extraction, MGWO as a feature selection, and the SVM as a classifier) has achieved better results compared to Alexnet, GoogleNet, and the SVM. The results showed in Table 10 give a comparison among the AlexNet, GoogleNet, SVM, and the proposed model through different metrics such as sensitivity, specificity, precision, F1-score, and accuracy. The proposed model achieved the highest accuracy in all datasets except the dataset named p2 in which the GoogleNet achieved the best accuracy. Figure 11 display the comparison among the different model concerning the accuracy metric. A comparison between the SVM which trained for the extracted features directly without feature selection and the SVM which trained to the selected features by the MGWO that extracted by AlexNet showed in Fig. 12. The ROC curve on the test set for the proposed model SVM is introduced in Fig. 13.

**Table 10:** Classification results for the four models.

Dataset	Sensitivity	Specificity	Precision	F1-Score	Accuracy
AlexNet					
P0	100	90.6	94.2	97.01	96.3
P1	92.3	95	96	94.11	93.5
P2	97.7	45.7	68.9	80.81	74.4
P3	100	100	100	100	100
P5	93.8	68.5	78.2	85.29	82.4
P6	82.3	93.3	93.3	87.46	87.5
P7	100	100	100	100	100
P9	74.5	56.7	61.2	67.2	65.2
P10	66.7	70	52.6	58.82	68.9
P11	84	90	91.3	87.5	86.7
GoogleNet					
P0	95.45	97.08	97.22	96.33	96.24
P1	98.1	82.5	87.9	92.72	91.3
P2	90.7	82.9	86.7	88.65	87.2
P3	100	96.7	92.6	96.16	97.6
P5	81.3	78.9	76.5	78.83	80
P6	85	84	81	82.95	84.4
P7	100	98.3	98.2	99.09	99.1
P9	89.1	73.3	75.4	81.68	80.9
P10	60	86.7	69.2	64.27	77.8
P11	80	90	90.9	85.1	84.4
SVM					
P0	94.74	96.15	96.43	95.58	95.41
P1	92.11	93.75	94.59	93.33	92.86
P2	86.84	83.33	84.62	85.71	85.14
P3	98.04	98.77	99.01	98.52	98.36
P5	80.68	79.07	79.78	80.23	79.89
P6	82.46	82.57	83.19	82.82	82.51
P7	96.91	97.83	97.92	97.41	97.35
P9	80.34	78.26	78.99	79.66	79.31
P10	77.39	76.19	78.07	77.73	76.82
P11	82.91	82.57	83.62	83.26	82.74
Proposed model					
P0	97.2	100	100	98.6	98.8
P1	94.2	97.5	98	96.06	95.7
P2	88.7	84	86.2	87.43	86.5
P3	100	100	100	100	100
P5	89.5	81.5	85	87.19	85.8
P6	95.1	94.1	95.1	95.1	94.7
P7	100	100	100	100	100
P9	81	100	100	89.5	91
P10	94.4	93.8	94.4	94.4	94.1
P11	87.9	96.4	96.7	92.09	91.8

## Conclusion and future work

We present in this paper a paradigm for the identification of plant diseases. Initially, a comparison is undertaken using the SVM, AlexNet, and Google Net-based transfer training method, which will be used on the edge servers with increased computational capability, to detect plant diseases. Then, with the AlexNet feature extraction and support vector machines for plant detection and classification diseases, we proposed a hybrid approach based on the modified gray wolf optimization algorithm for eliminating the resulted features from the AlexNet.

The proposed model can operate on Internet of Things (IoT) devices that use a framework that integrates fog and cloud computing with limited resources. Experimental evidence shows that the suggested models can detect plant diseases accurately using the minimum computational resources from real-world datasets. The proposed model worked better on most data sets. In the future, using blockchain technology, we hope to improve the fog environment without impacting the efficiency of features map extraction.

We will also develop apps to detect plant diseases to support smart agriculture with deep learning support.

## Data Availability

The datasets analyzed for this study available in “https://data.mendeley.com/datasets/hb74ynkjcn/1” focus on plants that contribute both ecologically and economically. All datasets used are open access data, and we didn’t use any private data. Our research complies with institutional, national, and international guidelines and legislation. We have permissions from our institutional committee for scientific research ethics to do this study and to collect plants data from the open access dataset. Throughout the entirety of the process of generating all the plots and statistics, Microsoft Excel 2016 and MATLAB were utilized. On the other hand, figures were generated using Microsoft PowerPoint and Adobe Photoshop. All plant samples used in the proposed model and tables are samples from the utilized dataset, which is open access data.

## Abbreviations

AI: Artificial intelligence
ML: Machine learning
DL: Deep learning
GWO: Grey wolf optimization
IoT: Internet of things
QoS: Quality of service
MGWO: Modified grey wolf optimization
DTL: Data transfer learning
SSD: Single shot detector
RCNN: Recurrent convolutional neural network
MLP: Multi-layer perceptron
FCN: Fully convolutional neural network
KNN: K-nearest neighbor
VGG: Visual geometry group
RF: Random forest
SVM: Support vector machines
CNN: Convolutional neural network
DCNN: Deep convolutional neural network
DAML: Deep adversarial metric learning
CAE: Convolutional autoencoder
GLDD: Grape leaf disease dataset
GPDCNN: Global pooling extended convolutional neural network
EPA: Environmental Protection Agency
TN: True negatives
FN: False negatives
FP: False positives
TP: True positives
ACO: Ant colony optimization
PSO: Particle swarm optimization
BOA: Butterfly optimization algorithm

## References

[1] Liakos KG, Busato P, Moshou D, Pearson S, Bochtis D (2018). Machine learning in agriculture: A review. Sensors.

[2] Xiao L, Zhao R, Zhang X (2020). Crop cleaner production improvement potential under conservation agriculture in China: A meta-analysis. J. Clean. Prod..

[3] Lee SH, Chan CS, Mayo SJ, Remagnino P (2017). How deep learning extracts and learns leaf features for plant classification. Pattern Recognit..

[4] Ale, L., Sheta, A., Li, L., Wang, Y., Zhang, N. Deep learning based plant disease detection for smart agriculture. In *2019 IEEE Globecom Workshops (GC Wkshps)*, 1–6 (2019).

[5] Liu J, Wang X (2021). Plant diseases and pests detection based on deep learning: A review. Plant Methods.

[6] Saleem MH, Potgieter J, Arif KM (2019). Plant disease detection and classification by deep learning. Plants.

[7] Singh V, Misra AK (2017). Detection of plant leaf diseases using image segmentation and soft computing techniques. Inf. Process. Agric..

[8] Mahum R (2023). A novel framework for potato leaf disease detection using an efficient deep learning model. Hum. Ecol. Risk Assess. Int. J..

[9] Gouse, S., Dulhare, U. N. Automation of Rice Leaf Diseases Prediction Using Deep Learning Hybrid Model VVIR. In *Advancements in Smart Computing and Information Security: First International Conference, ASCIS 2022, Rajkot, India, November 24–26, 2022, Revised Selected Papers, Part I*, 133–143 (2023).

[10] Sarhan, A. Fog computing as solution for IoT-based agricultural applications. In *Smart Agricultural Services Using Deep Learning, Big Data, and IoT*, 46–68 (IGI Global, 2021).

[11] Abbas A, Jain S, Gour M, Vankudothu S (2021). Tomato plant disease detection using transfer learning with C-GAN synthetic images. Comput. Electron. Agric..

[12] Thenmozhi K, Reddy US (2019). Crop pest classification based on deep convolutional neural network and transfer learning. Comput. Electron. Agric..

[13] Wiesner-Hanks T (2019). Millimeter-level plant disease detection from aerial photographs via deep learning and crowdsourced data. Front. Plant Sci..

[14] Too EC, Yujian L, Njuki S, Yingchun L (2019). A comparative study of fine-tuning deep learning models for plant disease identification. Comput. Electron. Agric..

[15] Chen T (2019). Intelligent identification system of disease and insect pests based on deep learning. China Plant Prot..

[16] Zhou G, Zhang W, Chen A, He M, Ma X (2019). Rapid detection of rice disease based on FCM-KM and faster R-CNN fusion. IEEE Access.

[17] Sethy PK, Barpanda NK, Rath AK, Behera SK (2020). Deep feature based rice leaf disease identification using support vector machine. Comput. Electron. Agric..

[18] Rahman CR (2020). Identification and recognition of rice diseases and pests using convolutional neural networks. Biosyst. Eng..

[19] Guo Y (2020). Plant disease identification based on deep learning algorithm in smart farming. Discret. Dyn. Nat. Soc..

[20] Atila Ü, Uçar M, Akyol K, Uçar E (2021). Plant leaf disease classification using EfficientNet deep learning model. Ecol. Inform..

[21] Gadekallu TR (2021). A novel PCA—whale optimization-based deep neural network model for classification of tomato plant diseases using GPU. J. Real-Time Image Process..

[22] Sanga, S., Mero, V., Machuve, D., Mwanganda, D. Mobile-based deep learning models for banana diseases detection. *arXiv Prepr. arXiv2004.03718* (2020).

[23] Chohan M, Khan A, Chohan R, Hassan S, Mahar M (2020). Plant disease detection using deep learning. Int. J. Recent Technol. Eng..

[24] Guo XQ, Fan TJ, Shu X (2019). Tomato leaf diseases recognition based on improved multi-scale AlexNet. Trans. Chin. Soc. Agric. Eng..

[25] Tan L, Lu J, Jiang H (2021). Tomato leaf diseases classification based on leaf images: A comparison between classical machine learning and deep learning methods. AgriEngineering.

[26] Agarwal M, Singh A, Arjaria S, Sinha A, Gupta S (2020). ToLeD: Tomato leaf disease detection using convolution neural network. Proc. Comput. Sci..

[27] Kundu N (2021). IoT and interpretable machine learning based framework for disease prediction in pearl millet. Sensors.

[28] Zhang S, Zhang S, Zhang C, Wang X, Shi Y (2019). Cucumber leaf disease identification with global pooling dilated convolutional neural network. Comput. Electron. Agric..

[29] Khamparia A, Saini G, Gupta D, Khanna A, Tiwari S, de Albuquerque VHC (2020). Seasonal crops disease prediction and classification using deep convolutional encoder network. Circuits Syst. Signal Process..

[30] Bedi P, Gole P (2021). Plant disease detection using hybrid model based on convolutional autoencoder and convolutional neural network. Artif. Intell. Agric..

[31] Faggella D (2020). Ai in agriculture—present applications and impact. Emerj Artif. Intell. Res. Insight..

[32] Boulent J, Foucher S, Théau J, St-Charles P-L (2019). Convolutional neural networks for the automatic identification of plant diseases. Front. Plant Sci..

[33] Samuel AL (1959). Some studies in machine learning using the game of checkers. IBM J. Res. Dev..

[34] Kujawa S, Mazurkiewicz J, Czekała W (2020). Using convolutional neural networks to classify the maturity of compost based on sewage sludge and rapeseed straw. J. Clean. Prod..

[35] Saleem MH, Potgieter J, Arif KM (2021). Automation in agriculture by machine and deep learning techniques: A review of recent developments. Precis. Agric..

[36] Weiss K, Khoshgoftaar TM, Wang D (2016). A survey of transfer learning. J. Big Data.

[37] Krizhevsky A, Sutskever I, Hinton GE (2012). Imagenet classification with deep convolutional neural networks. Adv. Neural Inf. Process. Syst..

[38] Hemmer M, Van Khang H, Robbersmyr KG, Waag TI, Meyer TJJ (2018). Fault classification of axial and radial roller bearings using transfer learning through a pretrained convolutional neural network. Designs.

[39] Dhillon A, Verma GK (2020). Convolutional neural network: A review of models, methodologies and applications to object detection. Prog. Artif. Intell..

[40] Özyurt F (2019). Efficient deep feature selection for remote sensing image recognition with fused deep learning architectures. J. Supercomput..

[41] Vapnik V (2013). The Nature of Statistical Learning Theory.

[42] Soman KP, Loganathan R, Ajay V (2009). Machine Learning with SVM and Other Kernel Methods.

[43] Pedersen, R., Schoeberl, M. An embedded support vector machine. In *2006 International Workshop on Intelligent Solutions in Embedded Systems*, 1–11 (2006).

[44] Altuntacs Y, Kocamaz F (2021). Deep feature extraction for detection of tomato plant diseases and pests based on leaf images. Celal Bayar Univ. J. Sci..

[45] Bishop, C. M. Pattern recognition. *Mach. Learn.* **128**(9) (2006).

[46] Mirjalili S, Mirjalili SM, Lewis A (2014). Grey wolf optimizer. Adv. Eng. Softw..

[47] Dereli S (2021). A new modified grey wolf optimization algorithm proposal for a fundamental engineering problem in robotics. Neural Comput. Appl..

[48] Tsipis A, Papamichail A, Angelis I, Koufoudakis G, Tsoumanis G, Oikonomou K (2020). An alertness-adjustable cloud/fog IoT solution for timely environmental monitoring based on wildfire risk forecasting. Energies.

[49] Guardo E, Di Stefano A, La Corte A, Sapienza M, Scatà M (2018). A fog computing-based iot framework for precision agriculture. J. Internet Technol..

[50] Goundar S, Bhushan SB, Rayani PK (2019). Architecture and Security Issues in Fog Computing Applications.

[51] Gómez-Chabla, R., Real-Avilés, K., Morán, C., Grijalva, P., Recalde, T. IoT applications in agriculture: A systematic literature review. In *2nd International Conference on ICTs in Agronomy and Environment*, 68–76 (2019).

[52] Srinivasan G, Vishnu Kumar N, Shafeer Ahamed Y, Jagadeesan S (2017). Providing smart agricultural solution to farmers for better yielding using IoT. Int. J. Adv. Sci. Eng. Res.

[53] Gao Z-M, Zhao J (2019). An improved grey wolf optimization algorithm with variable weights. Comput. Intell. Neurosci..

[54] What is Confusion Matrix and Advanced Classification Metrics? *Data Science and Machine Learning-blogger*. manisha-sirsat.blogspot.com (2019).

[55] Dheeru, E. D., Taniskidou, K. {UCI} Machine Learning Repository. (2017).

[56] Dwivedi S, Vardhan M, Tripathi S (2020). An effect of chaos grasshopper optimization algorithm for protection of network infrastructure. Comput. Netw..

[57] Abdel-Basset M, El-Shahat D, El-henawy I, de Albuquerque VHC, Mirjalili S (2020). A new fusion of grey wolf optimizer algorithm with a two-phase mutation for feature selection. Expert Syst. Appl..

[58] Gou J, Ma H, Ou W, Zeng S, Rao Y, Yang H (2019). A generalized mean distance-based k-nearest neighbor classifier. Expert Syst. Appl..

[59] El-Hasnony IM, Elhoseny M, Tarek Z (2022). A hybrid feature selection model based on butterfly optimization algorithm: COVID-19 as a case study. Expert Syst..

[60] Chouhan, S. S., Singh, U. P., Kaul, A., Jain, S. A data repository of leaf images: Practice towards plant conservation with plant pathology. In *2019 4th International Conference on Information Systems and Computer Networks (ISCON)*, 700–707 (2019).

